# Efficacy and Safety of *Salvia miltiorrhiza* for Treating Chronic Kidney Diseases: A Systematic Review and Meta-Analysis

**DOI:** 10.1155/2022/2117433

**Published:** 2022-06-14

**Authors:** Wei Zhang, Jun Li, Pan Yang, Gaoqiang Wang, Yanli Yue, Yuanfang Zhong, Hanyin Liu, Dingkun Gui, Youhua Xu, Niansong Wang

**Affiliations:** ^1^Macau University of Science and Technology, Faculty of Chinese Medicine, Taipa 999078, Macao, China; ^2^Department of Nephropathy, Shanghai Yangpu Hospital of TCM, Shanghai, China; ^3^Department of Nephropathy, Shanghai Jiao Tong University Affiliated Sixth People's Hospital, Shanghai, China; ^4^Macau University of Science and Technology, Zhuhai MUST Science and Technology Research Institute, Taipa 999078, Macao, China

## Abstract

**Objective:**

This meta-analysis evaluated the effects and potential harms of *Salvia miltiorrhiza* or its extracts Salvianolate and Tanshinone for the treatment of population with a chronic kidney disease (CKD).

**Methods:**

We searched for the randomized clinical trials (RCTs) through databases including the Cochrane Library, PubMed, Embase, Web of Science, Current Controlled Trials, China National Knowledge Infrastructure (CNKI), Wanfang Data Knowledge Service Platform (Wanfang Data), China Biology Medicine Disc (SinoMed), and Chinese Clinical Trial Registry (ChiCTR). Meta-analysis was performed with STATA 16 software after data extraction. The risk of bias was assessed with the Cochrane risk-of-bias tool (RoB 2.0), and the Grading of Recommendations Assessment, Development, and Evaluation (GRADE) framework was employed to evaluate the quality of evidence.

**Result:**

A total of 32 studies were included involving 2264 participants. Compared to the control group, the treatment group significantly decreased serum creatinine (SCr) (SMD −0.60, 95% CI −0.79 to −0.41, *P* < 0.0001), blood urea nitrogen (BUN) (SMD −0.66, 95% CI −0.81 to −0.50, *P* < 0.0001), Cystatin C (CysC) (SMD −5.16, 95% CI −14.84 to 4.53, *P*=0.297), 24 hour urine protein (24 h UPE) (SMD −0.70, 95% CI −1.21 to −0.19, *P*=0.008), time to initiation of dialysis (Log RR 0.43, 95% CI 0.23 to 0.81, *P*=0.0089), serum total cholesterol (TC) (SMD −0.53, 95% CI −0.88 to −0.17, *P*=0.0042, *P*=0.0035), plasma fibrinogen (FIB) (SMD −0.79, 95% CI −1.12 to −0.46, *P* < 0.0001), C-reactive protein (CRP) (SMD −0.56, 95% CI −0.93 to −0.19, *P*=0.0029); increased creatinine clearance (Ccr) (SMD 0.92, 95% CI 0.43 to 1.41, *P*=0.0002), glomerular filtration rate (GFR) (SMD 0.56, 95% CI 0.30 to 0.83, *P* < 0.001), effective rate (Log RR 0.30, 95% CI 0.23 to 0.37, *P* < 0.0001), and hemoglobin (Hb) (SMD 0.42, 95% CI 0.13 to 0.71, *P*=0.0042). Moreover, the incidences of adverse effects were similar between the two groups.

**Conclusions:**

*Salvia miltiorrhiza* or its extracts Salvianolate and Tanshinone, as a complementary therapy to conventional medicine, presents potential impacts to improve kidney functions and delay the progression of CKD without obvious adverse effects. However, the certainty of the evidence and the risk of bias are suboptimal and further clinical studies are still required to determine the underlying effects.

## 1. Introduction

A chronic kidney disease (CKD) arises from various heterogeneous diseases. The diagnosis of CKD rests on establishing a chronic reduction in the kidney function and structural damage.

The prevalence of CKD for stages 1–5 is 13.4% and 10.6% for stages 3–5 [1]. Contrary to diabetes or other metabolic diseases as prevalent as CKD, renal function impairment is often asymptomatic until very late stages [2]. According to the National Kidney Foundation, 30 million adults in the United States had CKD in 2017, and only 10 percent knew they had it, at a medical cost of $103 billion. In addition, CKD ranks fourteenth in the list of leading causes of death, which accounts for 12.2 deaths per 1,00,000 people, and the death rate of CKD will continue to increase to reach 14 per 1,00,000 people by 2030 [3]. In short, CKD has the characteristics of high incidence, high cost, high mortality, and low recognition rate.

Patients with CKD need efficient treatments to delay disease progression and improve the quality of life and the survival rate. In China, Chinese herbal medicines (CHMs) are widely used for the treatment of CKD. There are many prescriptions containing varieties or single CHM for CKD. It could also be said that every doctor's prescription may be different and is constantly modified during patients' followup. Hence, comparing the efficacy of diverse prescriptions is inherently heterogeneous and is not conducive to promotion outside China. Furthermore, according to the traditional Chinese medicine (TCM) theory, promoting blood circulation and removing blood stasis should be adopted throughout the treatment of CKD. *Salvia miltiorrhiza* (Danshen) is one of the most commonly used CHMs. Studies have demonstrated that *Salvia miltiorrhiza* is the top single CHM prescribed for CKD in China [4]. Medicinal parts of *Salvia miltiorrhiza* (Danshen) is the dried root and rhizomes of *Salvia miltiorrhiza* Bge. *Salvia miltiorrhiza* has specific preparations for clinical applications, such as *Salvia miltiorrhiza* tablet or injection and its extracts Salvianolate injection as well as Tanshinone injection, which all have strict quality control standards and the procedures are fully reproducible. *Salvia miltiorrhiza* and its extracts Salvianolate and Tanshinone are extensively used for CKD.

Clinically, a number of studies have displayed that *Salvia miltiorrhiza* can improve kidney function in CKD patients by increasing the glomerular filtration rate (GFR) and creatinine clearance (Ccr) and reducing serum creatinine (SCr) and proteinuria, but this conclusion is yet to be verified [5]. Research studies also reveal that *Salvia miltiorrhiza* could alleviate kidney injury via inhibiting oxidative stress and apoptosis [6] and exerting prominent renal protective effects in iron-overloaded mice by decreasing of iron deposition and suppression of lipid peroxidation and apoptosis in the kidney [7]. Tanshinone IIA significantly attenuates kidney fibrosis by inhibiting the recruitment of fibrocytes into the kidney [8] and decreases renal damage in diabetic rats via inhibiting oxidative stress and inflammation [9]. Salvianolate might alleviate the renal damage in chronic renal failure rats through antioxidant stress [10], accordingly attenuating glomerular injury, including albuminuria secretion, mesangial matrix expansion, foot process effacement in the kidneys of db/db mice, and ameliorated oxidative podocyte injury [11].

There is one previous meta-analysis that evaluated the efficacy and safety of Tanshinone for CKD [12], which includes 21 studies published before June 1, 2019. In our meta-analysis, we include *Salvia miltiorrhiza* and its extracts Salvianolate besides Tanshinone because they have a strong connection with each other and they are all widely used in China for CKD. All of the studies we included were published before November 9th, 2021. The subjects in the previous meta-analysis were diagnosed with diabetic nephropathy (3 studies), hypertensive renal damage (4 studies), renal vascular hypertension (1 study), and cardiorenal syndrome (1 study) rather than CKD (12 studies). Whereas, we focused on the subjects of patients diagnosed with CKD. The inclusion of subjects was more rigorous, whereas we may miss some patients with CKD who were diagnosed with hypertensive nephropathy or other diagnoses.

The current meta-analysis was performed to comprehensively evaluate the efficacy and safety of *Salvia miltiorrhiza* and its extracts Salvianolate and Tanshinone for the treatment of patients with CKD, with a view to provide substantial evidence for supporting its clinical application in CKD patients.

## 2. Methods

### 2.1. Protocol and Registration

This meta-analysis had been registered in PROSPEPO with registration number CRD42021291786.

### 2.2. Eligibility Criteria

#### 2.2.1. Types of Studies

All randomized controlled trials (RCTs) evaluating the efficacy of *Salvia miltiorrhiza* for CKD were included.

#### 2.2.2. Types of Participants

*(1) Inclusion Criteria*. Adults and children with CKD at all stages.

*(2) Exclusion Criteria*. Studies stating that participants had renal damage, but without baseline GFR, Ccr, or SCr; participants with diabetic nephropathy. These issues had been investigated in a previous study [13]; studies involving *Salvia miltiorrhiza* as one of several active components in a compound recipe were not included.

#### 2.2.3. Types of Interventions

Treatment group received *Salvia miltiorrhiza* or its extracts Salvianolate and Tanshinone. The control group received placebo, no treatment, or conventional treatment.

#### 2.2.4. Outcomes

*(1) Primary outcomes*. Kidney function measured by SCr, Ccr, GFR, blood urea nitrogen (BUN) cystatin C (CySC), or effective rate, proteinuria measured by 24 hour urinary protein excretion (24 h UPE), time to initiation of dialysis, and adverse effects.

*(2) Secondary outcomes*. Nutritional status assessed by serum albumin (ALB) and serum total cholesterol (TC), anemia measured by hemoglobin (Hb), hemorheology index measured by plasma fibrinogen (FIB), and inflammatory factor measured by C-reactive protein (CRP).

### 2.3. Search Methods

This meta-analysis was reported according to the Preferred Reporting Items for Systematic Reviews and Meta-Analyses (PRISMA 2020 [14,15]) (Supplementary Table S1). We searched the Cochrane Library, PubMed, Embase, Web of Science, Current Controlled Trials, and Chinese databases including China National Knowledge Infrastructure (CNKI), Wanfang Data Knowledge Service Platform (Wanfang Data), China Biology Medicine Disc (SinoMed), and Chinese Clinical Trial Registry (ChiCTR) from inception until November 9th, 2021 (Supplementary Table S2).

### 2.4. Study Selection

The search strategy described was used to obtain titles and abstracts of studies that may be relevant to this review. Titles, abstracts, and full texts were screened independently by two authors who determined which met the inclusion criteria and excluded studies that were not appropriate.

### 2.5. Data Collection Process

Data extraction was carried out independently by the same two authors using a pre-tested data extraction form. If more than one publication of one study existed, the publication with the most complete data was used. Any discrepancy between published versions was to be highlighted. Disagreements between authors were resolved by consensus and with a third author.

### 2.6. Quality Assessment and Statistical Methods

The publications included in this meta-analysis were subject to quality assessment according to the Cochrane criteria [15]. The risk of bias was assessed using the Cochrane risk-of-bias tool (RoB. 2.0) [16]. In addition, the Grading of Recommendations Assessment, Development, and Evaluation (GRADE) framework was employed to evaluate the quality of evidence contributing to each estimate [15].

The STATA 16 software was used for data analysis. For dichotomous outcomes, results were expressed as Log risk ratio (Log RR) with 95% confidence intervals (CI). For continuous outcomes, the standard mean difference (SMD) was presented with 95% confidence intervals (CI).

Heterogeneity was analyzed using a Chi^2^ test on *N* − 1 degrees of freedom, with an alpha of 0.1 used for statistical significance and with the *I*^2^-test [15]. *I*^2^ > 50% corresponds to high levels of heterogeneity, respectively. A subgroup or sensitivity analysis was conducted to explore the underlying causes of heterogeneity in treatment outcomes.

To assess small-study effects, we generated Egger's test or funnel plots [15] including at least 10 trials of varying size. If asymmetry was detected in the funnel plot, a contour-enhanced funnel plot was generated to assess whether the asymmetry was likely due to publication bias or other factors of the trials.

### 2.7. Additional Analyses

We conducted subgroup analyses to explore the impact of *Salvia miltiorrhiza* and its extracts Salvianolate and Tanshinone preparations.

## 3. Results

### 3.1. Study Selection

**Figure 1:**
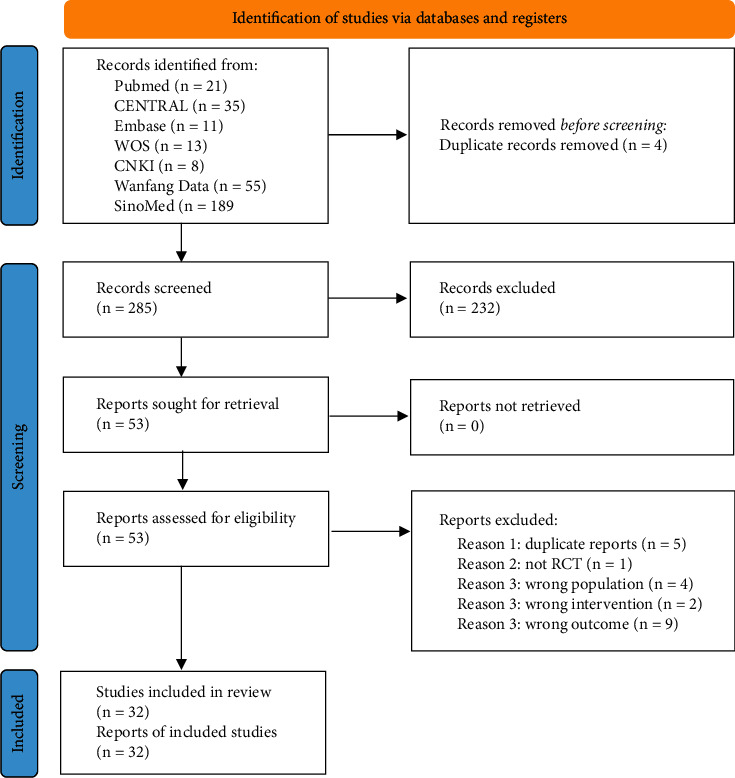
Flow of information through the different phases of the meta-analysis.

Our initial search found 332 records. After excluding 47 duplicate reports and 232 irrelevant records based on identification of titles and abstracts, we reviewed 53 full-text studies for inclusion and then 21 studies were further excluded. Finally, a total of 32 studies were included in the meta-analysis (Figure 1).

### 3.2. Study Characteristics

32 included studies involved 2264 participants and were conducted in hospitals of China and published in Chinese. All studies were parallel arm. Participants' age ranged from 18–96 years. Authors, year of publication, treatment plan, sample size, duration of treatment, and index of evaluation of each study are presented in Table 1.

### 3.3. Risk of Bias

**Figure 2:**
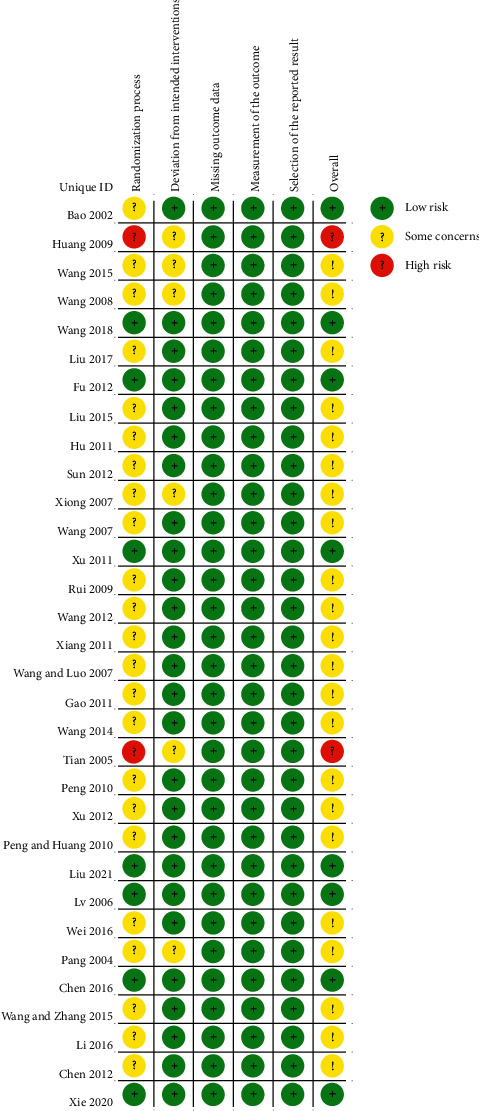
Risk of bias.

The 32 studies included were all RCTs, yet only 9 had detailed descriptions of the methods. In one study, computer-generated random numbers were used in the sequence generation process, and in 6 studies, random number tables were adopted. Nevertheless, in another 2 studies, patient record numbers were used. Only one study specified the method of blinding, and that was single-blind. All studies were assessed according to the RoB. 2.0 tool, of which 8 (25%) were assessed as “low risk of bias,” 22 (68.75%) as “some concerns,” and 2 (6.25%) as “high risk” (Figure 2).

### 3.4. Results of Included Studies

#### 3.4.1. Kidney Function

**Figure 3:**
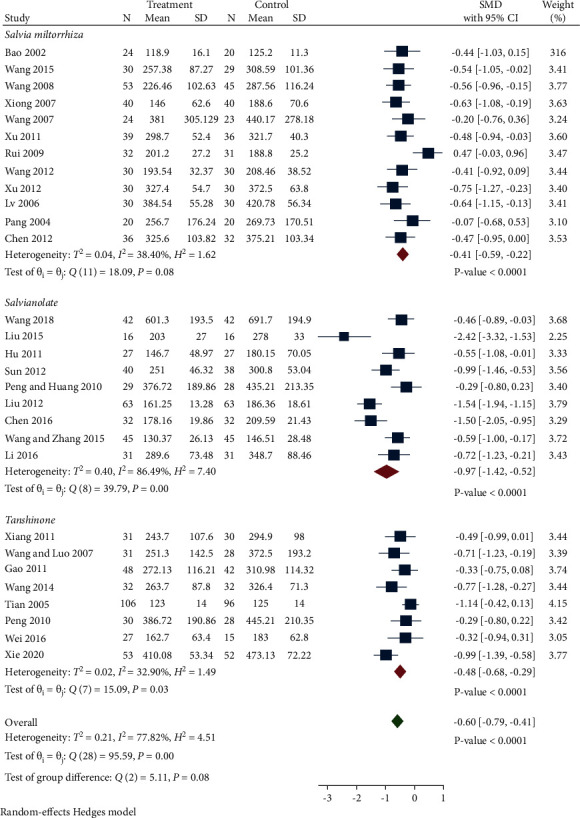
Forest plot of meta-analysis of SCr.

*(1) SCr*. A total of 29 studies compared Scr levels between the treatment and control group. We classified these studies into following subgroups based on the different preparations: *Salvia miltiorrhiza*, Salvianolate, and Tanshinone. As indicated in Figure 3, the random-effect model was used due to the high heterogeneity. Scr levels in the treatment group were significantly reduced compared with the control group (SMD −0.60, 95% CI −0.79 to −0.41, *P* < 0.0001, *I*^2^ = 77.82%). Subgroup analysis revealed that all of the 3 subgroups notably decreased SCr compared with control group (*Salvia miltiorrhiza* group: SMD −0.41, 95% CI −0.59 to −0.22, *P* < 0.0001, *I*^2^ = 38.4%. Salvianolate group: SMD −0.97, 95% CI −1.42 to −0.52, *P* < 0.0001, *I*^2^ = 80.49%. Tanshinone group: SMD −0.48, 95% CI −0.68 to −0.29, *P* < 0.0001, *I*^2^ = 32.90%). The Salvianolate group was the main source of heterogeneity. Then, we divided the studies of the Salvianolate group into various subgroups based on bias, when we removed the RCT of Wang et al. [21] which had the highest Scr and the Liu [24] with the smallest sample size, both low risk group and some concerns group had low heterogeneity (*I*^2^ = 0 and *I*^2^ = 6.03%).

Egger's test exhibited that there was no publication bias *P* < 0.001.

**Figure 4:**
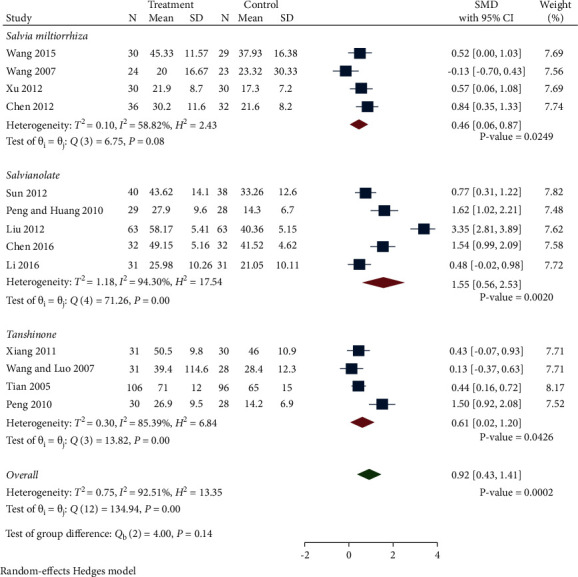
Forest plot of meta-analysis of Ccr.

*(2) Ccr*. A total of 13 studies compared Ccr levels between the treatment group and control group. We classified studies into different subgroups based on the preparations: *Salvia miltiorrhiza*, Salvianolate and Tanshinone. As demonstrated in Figure 4, the random-effect model was used because of the high heterogeneity. Ccr levels in the treatment group were significantly increased compared to the control group (SMD 0.92, 95% CI 0.43 to 1.41, *P*=0.0002, *I*^2^ = 92.51%). Subgroup analysis indicated that Ccr levels in the Salvianolate subgroups was distinctly increased compared with control group (SMD 1.55, 95% CI 0.56 to 2.53, *P*=0.002, *I*^2^ = 94.30%). Ccr levels in the *Salvia miltiorrhiza* and Tanshinone groups were increased but not significant compared with control group (SMD 0.46, 95% CI 0.06 to 0.87, *P* < 0.0249, *I*^2^ = 58.82%; SMD 0.61, 95% CI −0.02 to 1.20, *P*=0.0426, *I*^2^ = 85.39%).

Egger's test displayed that there was no publication bias (*P*=0.2624).

**Figure 5:**
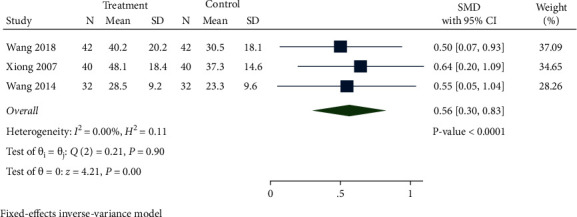
Forest plot of meta-analysis of GFR.

*(3) GFR.* A total of 3 studies compared GFR levels between the treatment group and control group. As shown in Figure 5, the heterogeneity was low (*I*^2^ = 0%) and the fixed-effect model was employed to analyze the data. GFR levels in the treatment group were significantly increased compared with the control group (SMD 0.56, 95% CI 0.30 to 0.83, *P* < 0.001, *I*^2^ = 0%).

**Figure 6:**
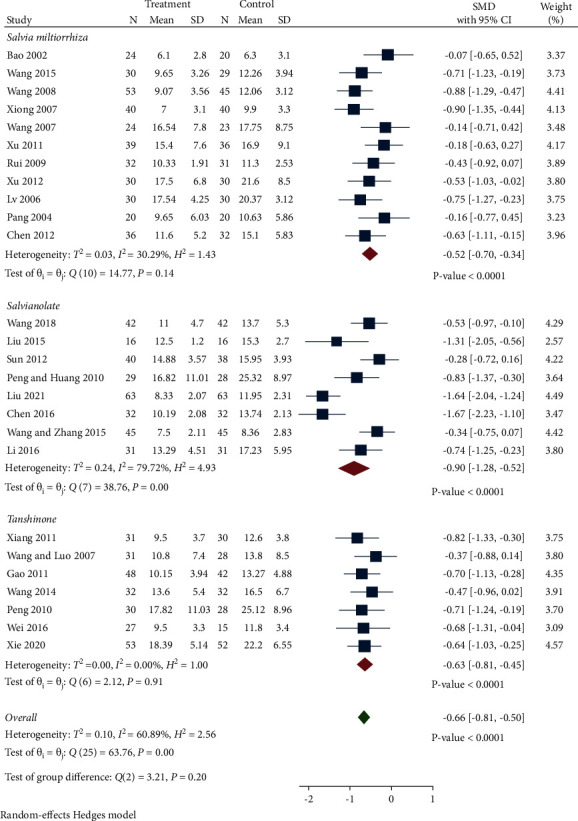
Forest plot of meta-analysis of BUN.

*(4) BUN*. A total of 26 studies reported BUN levels between the two groups. As presented in Figure 6, the random-effect model was used due to the high heterogeneity. BUN levels in the treatment group were significantly reduced compared with the control group (SMD −0.66, 95% CI −0.81 to −0.50, *P* < 0.0001, *I*^2^ = 60.89%). Subgroup analysis indicated that all of the 3 subgroups had decreased BUN compared with the control group (*Salvia miltiorrhiza* group: SMD −0.52, 95% CI −0.70 to −0.34, *P* < 0.0001, *I*^2^ = 30.29%; Salvianolate group: SMD −0.90, 95% CI −1.28 to −0.52, *P* < 0.0001, *I*^*2*^ = 79.72%; Tanshinone group: SMD −0.63, 95% CI −0.81 to −0.45, *P* < 0.0001, *I*^*2*^ = 0%). The Salvianolate group was the main cause of heterogeneity.

Egger's test reflected that no publication bias existed (P=0.9602).

**Figure 7:**
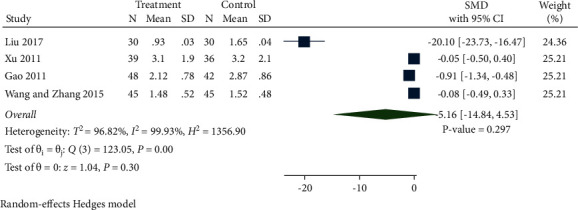
Forest plot of meta-analysis of CysC.

*(5) CysC.* A total of 4 studies recorded CysC levels between the treatment group and control group. As presented in Figure 7, CysC levels in the treatment group were decreased but without significance compared with the control group (SMD −5.16, 95% CI −14.84 to 4.53, *P*=0.297, *I*^2^ = 99.93%).

**Figure 8:**
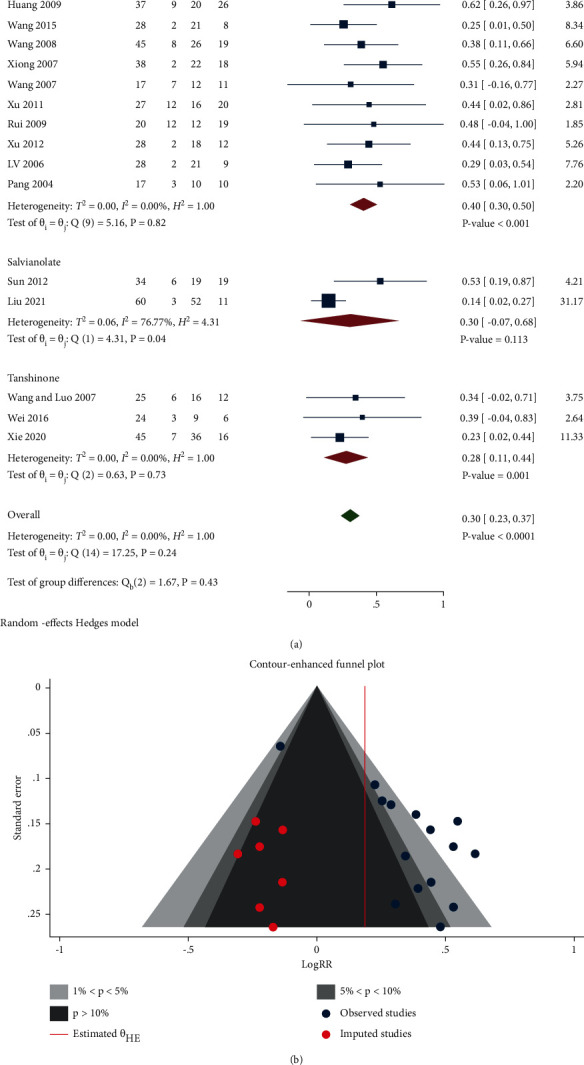
(a) Forest plot of meta-analysis of effective rate. (b) Contour-enhanced funnel plot of meta-analysis of the effective rate.

*(6) Effective Rate.* A total of 15 studies compared the effective rate between the two groups. As listed in Figure 8(a), the effective rate in the treatment group was significantly higher compared with the control group (Log RR 0.30, 95% CI 0.23 to 0.37, *P* < 0.0001, *I*^2^ = 0%), and the heterogeneity was low. Subgroup analysis indicated that the effective rates were remarkably higher in *Salvia miltiorrhiza* and Tanshinone groups compared to the control group (Log RR 0.40, 95% CI 0.30 to 0.50, *P* < 0.0001, *I*^2^ = 0%; Log RR 0.28 95% CI 0.11 to 0.44, *P* < 0.001, *I*^2^ = 0%), the effective rate was higher in the Salvianolate group compared with the control group (Log RR 0.30, 95% CI -0.07 to 0.68, *P*=0.113, *I*^2^ = 76.77%). Liu et al. [40] used alprostadil injection combined Salvianolate injection in the treatment group, which might bias the heterogeneity. Furthermore, removal of this study resulted in a considerable reduced *I*^2^ (*I*^2^ = 0%).

Egger's test revealed that there was publication bias (*P* < 0.05). As shown in Figure 8(b), 7 studies were imputed and 2 studies were in the area of 5% <*P* < 10%.

#### 3.4.2. 24 h UPE

**Figure 9:**
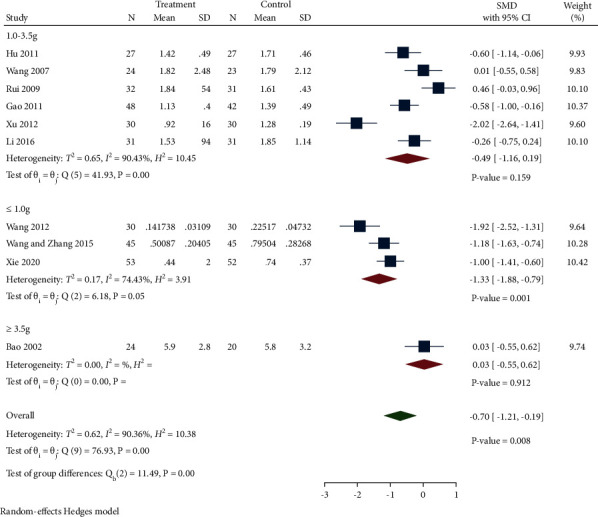
Forest plot of meta-analysis of 24 h UPE.

A total of 10 studies compared 24 h UPE levels between the treatment group and control group. As suggested in Figure 9, 24 h UPE levels in the treatment group were observably reduced compared with the control group (SMD −0.70, 95% CI −1.21 to −0.19, *P*=0.008, *I*^2^ = 90.36%). We classified these studies into three subgroups based on the degrees of 24 h UPE: ≤1.0 g, 1.0–3.5 g, ≥3.5 g. Subgroup analysis indicated that the effect of *Salvia miltiorrhiza* was inversely proportional to the degree of proteinuria. (SMD −11.33, 95% CI −1.88 to −0.79, *P* < 0.0001, *I*^*2*^ = 74.43%; SMD −0.49, 95% CI −1.16 to −0.19, *P*=0.159, *I*^*2*^ = 90.43%; SMD 0.03, 95% CI −0.55 to 0.62, *P*=0.912).

Egger's test hinted that there was no publication bias (*P*=0.5167).

#### 3.4.3. Time to Initiation of Dialysis

**Figure 10:**
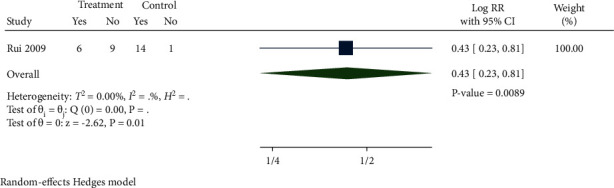
Forest plot of meta-analysis of time to initiation of dialysis.

There was only one study [23] that reported the time to initiation of dialysis. As demonstrated in Figure 10, in comparison with the control group, there were less CKD patients into initiation of dialysis in the treatment group (Log RR 0.43, 95% CI 0.23 to 0.81, *P*=0.0089).

#### 3.4.4. Adverse Effects

**Figure 11:**
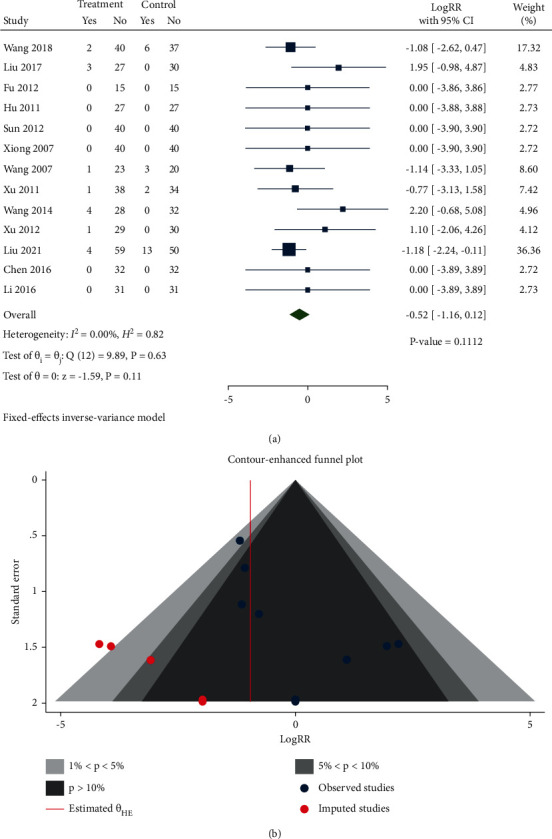
(a) Forest plot of meta-analysis of adverse effects. (b) Contour-enhanced funnel plot of meta-analysis of adverse effects.

In all, 13 studies reported adverse effects. As presented in Figure 11(a), adverse effects in the treatment group did not differ significantly from that of the control group (Log RR −0.52, 95% CI: −1.16 to 0.12, *P*=0.1112, *I*^2^ = 0%).

Egger's test indicated that there was publication bias *P*=0.0349. As shown in Figure 11(b), 7 studies were imputed, 2 studies were in the area of 1% < *P* < 5%, and one study was in the area of 5% < *P* < 10%.

#### 3.4.5. Nutritional Status

**Figure 12:**
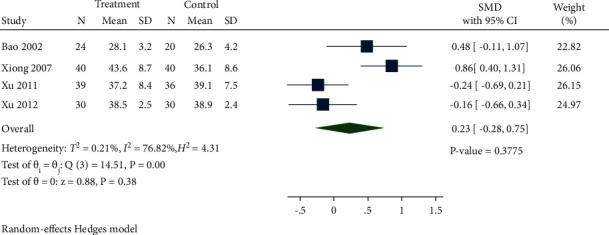
Forest plot of meta-analysis of ALB.

*(1) ALB*. A total of 4 studies documented ALB levels between the treatment group and control group. As displayed in Figure 12, there was no significance between the *Salvia miltiorrhiza* group and control group (SMD 0.23, 95% CI −0.28 to 0.75, *P*=0.3775, *I*^2^ = 76.82%).

**Figure 13:**
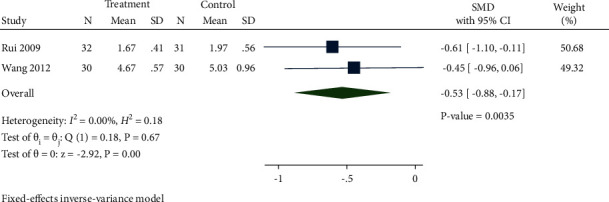
Forest plot of meta-analysis of TC.

*(2) TC.* A total of 2 studies recorded TC levels between the treatment group and control group. As expressed in Figure 13, TC levels in the treatment group were distinctly reduced compared with the control group (SMD −0.53, 95% CI−0.88 to −0.17, *P*=0.0035, *I*^2^ = 0%).

#### 3.4.6. Anemia Measured: Hb

**Figure 14:**
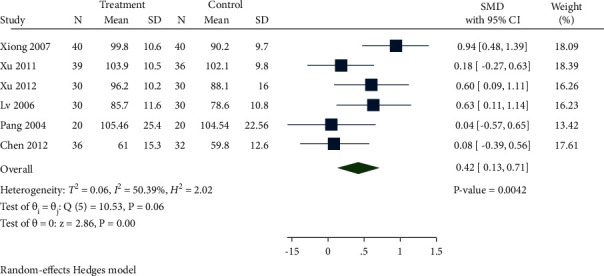
Forest plot of meta-analysis of Hb.

A total of 6 studies reported HB levels. As manifested in Figure 14, Hb levels in the treatment group were significantly enhanced compared with the control group (SMD 0.42, 95% CI 0.13 to 0.71, *P*=0.0042, *I*^2^ = 50.39%).

#### 3.4.7. Hemorheology Index: FIB

**Figure 15:**
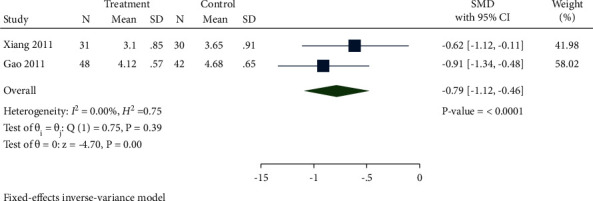
Forest plot of meta-analysis of FIB.

A total of 2 studies had data of FIB levels. As presented in Figure 15, FIB levels in the treatment group were significantly reduced compared with the control group (SMD −0.79, 95% CI −1.12 to −0.46, *P* < 0.0001, *I*^2^ = 0%).

#### 3.4.8. Inflammatory Factor: CRP

**Figure 16:**
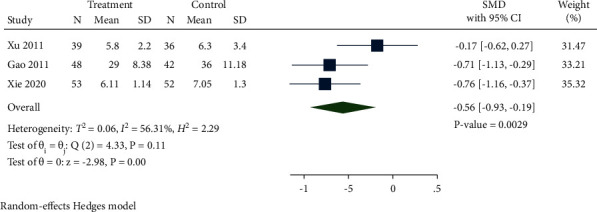
Forest plot of meta-analysis of CRP.

A total of 3 studies compared CRP levels between the treatment group and control group. As displayed in Figure 16, CRP levels in the treatment group were notably reduced compared with the control group (SMD −0.56, 95% CI −0.93 to −0.19, *P*=0.0029, *I*^2^ = 56.31%).

### 3.5. Certainty of Evidence

All outcome indicators were evaluated by GRADEpro GDT. The quality of evidence was downgraded for the risk of bias or publication bias. After comprehensive analysis, the summary table was formed, and it was found that 2 outcome indicators (14.29%) were of high quality, 12 outcome indicators (71.43%) were of moderate quality, and 2 outcome indicators (14.29%) were of low quality (Supplementary Table S3).

### 3.6. Publication Bias

Egger's test declared that no publication bias in the indicators of SCr, CCr, BUN, and 24 h UPE were observed. Whereas, there was publication bias in the indicator of the effective rate; the contour-enhanced funnel plot suggested that 7 studies were imputed and 2 studies were in the area of 5% < *P* < 10%. In the indicator of adverse effects, Egger's test hinted that there existed a publication bias, 7 studies were imputed, 2 studies were in the area of 1% < *P* < 5%, and another one study was in the area of 5% < *P* < 10%.

## 4. Discussion

In this meta-analysis, we intended to explore potential effects of *Salvia miltiorrhiza* for people with CKD on disease progression and complications. 32 studies that involved 2264 participants with CKD were included. In the aspects of kidney functions (SCr, Ccr, GFR, BUN, CySC, and effective rate), the comparison results revealed that the treatment group significantly reduced SCr, BUN and CysC, increased Ccr, GFR, and effective rate, indicating that this complementary therapy may have good effects for kidney functions. Proteinuria is a common clinical feature in patients with CKD, which is also an important factor in the CKD progression. The comparison demonstrated that the treatment group significantly reduced 24 h UPE. Furthermore, subgroup analysis indicated that the effect was inversely proportional to the degree of proteinuria, which confirmed that this complementary therapy may delay the CKD progression. In one study, time to initiation of dialysis was observed, the comparison uncovered that there were less CKD patients into initiation of dialysis in the treatment group. Unfortunately, the sample size of this study was too small (*n* = 30). 13 studies reported adverse effects. The incidence of adverse effects was similar between the two groups. Meanwhile, some indicators of complications were compared. The treatment group alleviated CKD-associated complications, and the complementary therapy may have effects on reducing TC, FIB, and CRP levels and increasing Hb levels.

According to the TCM theory, *Salvia miltiorrhiza* (Danshen) is one of the most commonly used CHMs with the effect of promoting blood circulation to remove blood stasis, which should be used throughout the treatment of CKD. Studies have indicated that *Salvia miltiorrhiza* is the top single CHM prescribed for the treatment of CKD in China. A number of studies have testified that *Salvia miltiorrhiza* can improve kidney functions in CKD patients by increasing GFR and Ccr and reducing SCr and proteinuria [4]. Research studies also revealed that *Salvia miltiorrhiza* alleviated kidney injury via inhibiting oxidative stress and apoptosis [5]. Tanshinone IIA obviously attenuated kidney fibrosis by inhibiting the recruitment of fibrocytes into the kidney [7]. Besides, Salvianolate alleviated the renal damage and attenuated glomerular injury through antioxidant stress [9].

There are certain limitations of the evidence that should be considered. According to the RoB 2.0 tool, 75% of the studies were assessed as “some concerns” or “high risk.” Although all of the included studies claimed to have a randomized controlled design, but only 9 had detailed descriptions of the methods. Methodological deficiencies are related to the lack of a clear description of randomization, allocation concealment, and binding. In 7 studies, computer-generated random numbers or random number tables were used. Whereas, patient record numbers were used in 2 studies. Only one study specified the method of blinding, whereas, that was single-blind. Egger's test implied that there was publication bias in the indicator of effective rate and adverse effects.

In addition, the heterogeneity was high in the results of some indicators. As for SCr, Salvianolate group was the main cause of heterogeneity. When we removed the RCT of Wang et al. [21] with the highest baseline Scr and the Liu et al. [24] with the smallest sample size, the heterogeneity significantly declined. In the indicator of the effective rate, Liu et al. [40] used alprostadil injection combined with Salvianolate injection for the treatment group, which could bias the heterogeneity. These abovementioned statements indicate that we should expand the sample size, to improve the quality of RCT. Meanwhile, limited by language barriers, only Chinese and English databases were searched, and all the included studies were conducted in China, which might affect the final results to a certain degree. Hence, there exist doubts about the applicability of evidence in other countries.

## 5. Conclusions

Current evidences indicate that *Salvia miltiorrhiza* may have certain benefits for CKD patients as a complementary therapy, which could improve kidney functions, reduce proteinuria, delay the progression of CKD, and improve several complications resulting from CKD. However, the certainty of the evidence and the risk of bias are suboptimal and further clinical studies are still needed to determine the potential effects of *Salvia miltiorrhiza* for patients with CKD.

## Figures and Tables

**Table 1:** Characteristics of included studies.

Study Year	Treatment	Control	Number (T/C)	Duration	Results report
Bao et al. 2002 [17]	*Salvia miltiorrhiza* injection (6–10 ml ivgtt qd) + conventional steroid treatment	Conventional steroid treatment	24/20	2 weeks	Scr, BUN, 24 h UPE, ALB
Huang 2009 [18]	*Salvia miltiorrhiza* injection (20 ml ivgtt qd) + conventional treatment	Conventional treatment	46/46	2 weeks	Effective rate
Wang et al. 2015 [19]	*Salvia miltiorrhiza* Injection (1 ml ivgtt qd) + Huang qi injection (2.5 ml) + conventional treatment	Conventional treatment	30/29	2 months	Scr, BUN, Ccr
Wang and Zhao [20]	*Salvia miltiorrhiza* injection (400 mg ivgtt qd) + conventional treatment	Conventional treatment	53/45	35 days	Effective rate, SCr
Wang et al. 2008 [21]	Salvianolate injection (200 mg ivgtt qd) + alprostadil Injection (30 *μ*g ivgtt qd) + reduced glutathione injection (2.4 g ivgtt qd) conventional treatment	Conventional treatment	42/42	7 days	BUN SCr, BUN, GFR
Liu 2017 [22]	Salvianolate injection (200 mg ivgtt qd) + conventional treatment	Conventional treatment	30/30	2 weeks	CySC, adverse effect
Fu et al. 2012 [23]	Salvianolate Injection (200 mg ivgtt qd) + alprostadil Injection (20 *μ*g ivgtt qd) + reduced glutathione Injection (2.4 g ivgtt qd) + conventional treatment	Conventional treatment	15/15	12 to 64 months	Effect time to initiation of
Liu 2015 [24]	Salvianolate injection (100 mg ivgtt qd) + conventional treatment	Conventional treatment	16/16	2 weeks	Dialysis, adverse effect of SCr, BUN
Hu et al. 2011 [25]	Salvianolate injection (200 mg ivgtt qd) + conventional treatment	Conventional treatment	27/27	4 weeks	SCr, 24 h UPE
Sun and Luo 2012 [26]	Salvianolate injection (200 mg ivgtt qd) + conventional treatment	Conventional treatment	40/38	2 weeks	Effective rate, SCr
Xiong et al. 2007 [27]	*Salvia miltiorrhiza* injection (80 mg ivgtt qd) + TCM decoction ((Rhubarb 20 g, calcined keel 20 g, calcined oyster 20 g, dandelion 20 g, Ligusticum chuanxiong 20 g) 300 ml retention enema for 1-2 h, qd) + conventional treatment	Conventional treatment	40/40	30 days	BUN, Ccr, adverse effect effective rate, SCr
Wang et al. 2007 [28]	*Salvia miltiorrhiza* injection (800 mg ivgtt qd) + conventional treatment	Xueshuantong Injection (450 mg ivgtt qd) + conventional treatment	24/23	15 days	BUN, GFR, Hb, ALB, adverse effect effective rate
Xu et al. 2011 [29]	*Salvia miltiorrhiza injection (20 ml ivgtt qd) + Huang qi injection (40 g ivgtt qd) + conventional treatment*	Conventional treatment	39/36	2 weeks	Effective rate, SCr
Guohua et al. 2009 [30]	*Salvia miltiorrhiza* tablet (4 pills tid p.o.) + irbesartan (150 mg qd p.o.) + conventional treatment	Irbesartan (150 mg qd pop.o.) + conventional treatment	32/31	6 months	BUN, CysC, Hb, ALB, CRP effective rate, SCr
Wang 2012 [31]	Salvia miltiorrhiza injection (800 mg ivgtt qd) + Valsartan (80 mg p. o. qd) + conventional treatment	Valsartan (80 mg p.o. qd) + conventional treatment	30/30	4 weeks	BUN, 24 h UPE, TC SCr, 24 h UPE, TC
Xiang and Mo 2011 [32]	Sodium Tanshinone II A sulfonate injection (40 mg ivgtt qd) + TCM decoction ((Rhubarb 10 g, Calcined oyster 20 g, Dandelion 30 g) 400 ml retention enema for 20–30 m, bid) + conventional treatment	TCM decoction [(Rhubarb 10 g, Calcined oyster 20 g, Dandelion 30 g) 400 ml retention enema for 20–30 m, bid] + conventional treatment	31/30	8 weeks	SCr, BUN, Ccr
Wang and Xian-Qin 2007 [33]	Sodium Tanshinone II A sulfonate Injection (20 ml ivgtt qd) + conventional treatment	Conventional treatment	31/28	2 months	FIB effective rate, SCr
Gao and Gao 2011 [34]	Sodium Tanshinone II A sulfonate injection (50 mgivgtt qd) + conventional treatment	Conventional treatment	48/42	4 weeks	BUN, Ccr SCr, BUN, 24 h
Wang 2014 [35]	Sodium Tanshinone II A sulfonate injection (40 mg ivgtt qd) + Alprostadil injection (10 *μ*g ivgtt qd) + conventional treatment	Conventional treatment	32/32	28 days	UPE, CySC, CRP, FIB SCr, BUN, GFR
Tian et al. 2005 [36]	*Salvia miltiorrhiza* injection (60 ml ivgtt qd) + conventional treatment	Conventional treatment	106/96	10 days	Adverse effect SCr, Ccr
Peng et al. 2010 [37]	Sodium tanshinone II A sulfonate injection (50 mg ivgtt qd) + Haikunshenxi capsule (2 pills p.o. tid) + conventional treatment	Conventional treatment	30/28	3 weeks	SCr, BUN, Ccr
Xu et al. 2012 [38]	*Salvia miltiorrhiza* injection (1200 mg ivgtt qd) + Haikunshenxi capsule (2 pills p.o. tid) + conventional treatment	Conventional treatment	30/30	15 days	Effective rate, SCr,BUN, 24 h, UPE, ALB, Ccr, Hb
Peng et al. 2010 [39]	Salvianolate injection (200 mg ivgtt qd) + Alprostadil	Conventional treatment	29/28	2 weeks	SCr, BUN, Ccr
Liu et al. [40]	Salvianolate injection (100 mg ivgtt qd) + alprostadil injection (20 *μ*g ivgtt qd) + conventional treatment	Alprostadil injection (20 *μ*givgtt qd) + conventional treatment	63/63	4 weeks	Effective rate, SCr,BUN, Ccr
Lv et al. 2006 [41]	*Salvia miltiorrhiza* tablet (4 pills tid p.o.) + Jieduxiezhuo II decoction 150 ml retention enema qd + conventional treatment	Jieduxiezhuo II decoction 150 ml retention enema qd + conventional treatment	30/30	1 month	Effective rate, SCr, BUN, Hb
Guowei et al. 2016 [42]	Sodium Tanshinone II A sulfonate injection (40 mg ivgtt qd) + alprostadil injection (10 *μ*g ivgtt qd) + conventional treatment	Conventional treatment	27/15	2 weeks	Effective rate, SCr
Pang 2004 [43]	*Salvia miltiorrhiza* injection (30 ml ivgtt qd) + TCM decoction 200 ml retention enema bid + low molecular levo-anhydride injection (250 ml ivgtt qd) + conventional treatment	Conventional treatment	20/20	12	BUN, effective rate, SCr
Chen 2016 [44]	Salvianolate Injection (150 mg ivgtt qd + conventional treatment	Conventional treatment	32/32	14 days	SCr, BUN, Ccr
Wang 2015 [45]	Salvianolate injection (100 mg ivgtt qd) + Valsartan (80 mg p.o. qd) + conventional treatment	Valsartan (80 mg p.o. qd) + conventional treatment	45/45	2 weeks	SCr, BUN, 24 h UPE, CySC
Zhi et al. 2016 [46]	Salvianolate injection (200 mg ivgtt qd) + Shenshuaining granules (1 bag p.o. 3–4 times/day) + conventional treatment	Conventional treatment	31/31	2 weeks	SCr, BUN, Ccr, adverse effect
Chen and Lu 2012 [47]	Salvia miltiorrhiza injection (0.8 g ivgtt qd) + sodium ferulate injection (0.3 g ivgtt qd) + conventional treatment	Conventional treatment	36/32	5 weeks	Effective rate, SCr, BUN, Ccr, Hb
Xie et al. 2020 [48]	Sodium tanshinone II A sulfonate injection (50 mg ivgtt qd) + Corbrin capsule (2 g p.o. tid) + conventional treatment	Corbrin capsule (2 g p.o.tid) + conventional	53/52	2 months	Effective rate, SCr, BUN, 24 h UPE

T. treatment group; C. control group; p.o.: per os; qd: quaque die; tid: ter in die; ivgtt: injectio intiavenosus gutta; 24 h UPE: 24 hour urine protein excretion; GFR: glomerular filtration rate; Ccr. creatinine clearance; SCr: serum creatinine; BUN: Blood urea nitrogen; CysC. cystatin C; ALB: serum albumin; TC: serum total cholesterol; Hb: haemoglobin; FIB: plasma fibrinogen; and CRP: C-reactive protein.

## Data Availability

The data used to support the findings of this study are available on request from the first and corresponding authors.

## Supplementary Materials

Supplementary Materials Table S1: PRISMA 2020 Checklist. Table S2: search strategies. Table S3: certainty of Evidence evaluated by GRADEpro GDT. The original contributions presented in the study were included in the article/Supplementary Material; further inquiries could be directed to the first author.

## References

[1] Hill N. R., Fatoba S. T., Oke J. L. (2016). Global prevalence of chronic kidney disease-a systematic review and meta-analysis. *PLoS One*.

[2] Bellasi A., Di Lullo L., Di Iorio B. (2019). Chronic kidney disease: the silent epidemy. *Journal of Clinical Medicine*.

[3] World Health Organization (2016). Mortality and global health estimates: causes of death, projections for 2015–2030; projection of death rates. https://apps.who.int/gho/data/node.main.%20PROJRATEWORLD?lang=en.

[4] Huang K.-C., Su Y.-C., Sun M.-F., Huang S.-T. (2018). Chinese herbal medicine improves the long-term survival rate of patients with chronic kidney disease in taiwan: a nationwide retrospective population-based cohort study. *Frontiers in Pharmacology*.

[5] Sun J., LA Y. (2010). Pharmacological effects of Salvia miltiorrhiza and its components and the latest application progress in kidney disease. *Chinese Journal of Integrated Traditional and Western Medicine Nephropathy*.

[6] Wang X., Liu W., Jin G. (2022). Salvia miltiorrhiza polysaccharides alleviates florfenicol induced kidney injury in chicks via inhibiting oxidative stress and apoptosis. *Ecotoxicology and Environmental Safety*.

[7] Guan S., Ma J., Zhang Y. (2013). Danshen (Salvia miltiorrhiza) injection suppresses kidney injury induced by iron overload in mice. *PLoS One*.

[8] Jiang C., Shao Q., Jin B., Gong R., Zhang M., Xu B. (2015). Tanshinone IIA attenuates renal fibrosis after acute kidney injury in a mouse model through inhibition of fibrocytes recruitment. *Bio-Medical Research International*.

[9] Chen X., Wu R., Kong Y. (2017). Tanshinone II Aattenuates renal damage in STZ-induced diabetic rats via inhibiting oxidative stress and inflammation. *Oncotarget*.

[10] Zhang G., Cui G., Tong S., Cao Q. (2019). Salvianolic acid a alleviates the renal damage in rats with chronic renal failure. *Acta Cirurgica Brasileira*.

[11] Liang Y., Liu H., Fang Y. (2021). Salvianolate ameliorates oxidative stress and podocyte injury through modulation of NOX4 activity in db/db mice. *Journal of Cellular and Molecular Medicine*.

[12] Zhou Y., Jiang S. M., Li L. (2020). Efficacy and safety of tanshinone for chronic kidney disease: a meta-analysis. *Evidence Based Complement Alternative Medicine*.

[13] Shen Y., Wang S., Liu Y. (2020). The effects of salvianolate combined with western medicine on diabetic nephropathy: a systematic review and meta-analysis. *Frontiers in Pharmacology*.

[14] Page M. J., McKenzie J. E., Bossuyt P. M. (2021). The PRISMA 2020 statement: an updated guideline for reporting systematic reviews. *BMJ*.

[15] Page M. J., Moher D., Bossuyt P. M. (2021). PRISMA 2020 explanation and elaboration: updated guidance and exemplars for reporting systematic reviews. *BMJ*.

[16] Sterne J. A. C., Savović J., Page M. J. (2019). RoB 2: a revised tool for assessing risk of bias in randomised trials. *BMJ*.

[17] Bao H., Yu H., Wang L., Long M., Chen R. (2002). Effects of salvia miltiorrhiza injection on plasma endothelin and soluble interleukin-2 receptor in children with primary nephrotic syndrome. *Chinese Journal of Integrated Traditional and Western Medicine*.

[18] Huang R. (2009). Clinical efficacy analysis of 46 cases of salvia miltiorrhiza injection in treatment of chronic renal failure. *Chinese and foreign Medicine*.

[19] Wang B., Mi J., Zheng Y., Tie L. (2015). Effect of salvia miltiorrhiza and Astragalus injection combined with acupoint injection on 30 cases of chronic renal failure. *National Physician BBS*.

[20] Wang N., Zhao R. (2008). Effect of salvia miltiorrhiza lyophilized powder needle on delaying the progression of chronic renal failure. *Chinese Journal of Modern Integrated Traditional and Western Medicine*.

[21] Wang Y., Chen X., Zhao H. (2018). Effect of salvianolate, alprostl and glutathione combined therapy on eGFR level in patients with chronic renal failure. *Shaanxi Medical Journal*.

[22] Liu Y. B. (2017). Effect of salvianolate on cystatin C in chronic renal failure. *Henan Province Traditional Chinese Medicine*.

[23] Fu P., Huang X., Yuan A., Yu G., Mei X., Cui R. (2012). A randomized controlled study of salvianolate combined with alprostol and glutathione to delay renal function decline in patients with chronic kidney disease. *Chinese Journal of Integrated Traditional and Western Medicine*.

[24] Liu Y. (2015). Salvianolate in the treatment of 16 cases of chronic renal failure. *China Traditional Chinese Medicine Science and Technology*.

[25] Hu Y., Yan Z. H., Yan X. (2011). Effects of salvianolate on plasma ET-1 and CGRP levels in early chronic renal failure. *Guizhou Pharmaceutical*.

[26] Sun W., Luo Z. (2012). Salvia miltiorrhiza polyphenols middle acid salt treatment of chronic renal failure clinical observation. *Journal of Gansu Medicine*.

[27] Xiong Y., He T., Jin L. (2007). Effect of salvia miltiorrhiza injection combined with Chinese traditional medicine enema on chronic renal failure. *Chinese Journal of Traditional Chinese Medicine Emergency*.

[28] Wang Y., Du J., Li C. Clinical observation of Salvia Miltiorrhiza powder in treatment of stage 2–4 chronic kidney disease in 47 cases.

[29] Xu G., Yan L., Yuan L., Xie P., Chen Y., Zhao J. (2011). Effects of salvia miltiorrhiza and Astragalus injection on acute kidney injury based on chronic kidney disease. *Chinese Journal of Clinical Physicians (Electronic Edition)*.

[30] Guohua R., Yao G., Pan R., Wu X. (2009). Efficacy of salvia miltiorrhiza combined with irbesartan in the treatment of early chronic renal failure. *Chinese Journal of Modern Integrated Traditional and Western Medicine*.

[31] Wang H. Q. (2012). Efficacy evaluation of salvia miltiorrhiza combined with valsartan in the treatment of early chronic renal failure. *Hainan Medical Journal*.

[32] Xiang Q. I., Mo Z. (2011). Tanshinone II A sodium sulfonate combined with Traditional Chinese medicine enema in the treatment of chronic renal failure. *Journal of Clinical Nephrology*.

[33] Wang H., Xian-Qin L. (2007). Clinical study of tanshinone II A sodium sulfonate injection in the treatment of chronic renal failure azotemia stage. *Modern Health care Medical Innovation Research*.

[34] Gao H., Gao Y. (2011). Clinical observation of tanshinone II A sodium sulfonate injection in treatment of chronic renal failure. *Shandong Medicine*.

[35] Wang W. (2014). Effect of tanshinone combined with alprostadil on left ventricular hypertrophy in patients with chronic renal failure. *Journal of Shandong Medical College*.

[36] Tian X., Xue W., Ding X. (2005). Application of Danshen injection on early stage of renal transplantation. *Chinese Journal of Integrated Traditional and Western Medicine*.

[37] Peng W., Huang Y., Huang D., Wang S. (2010). Efficacy of Haikun Shenxi capsule combined with tanshinone II A sodium sulfonate in the treatment of chronic renal failure. *Modern Chinese Medicine Application*.

[38] Xu H., Wang Q., Gao X., Huang M., Chen L. (2012). Clinical analysis of Haikun Shenxi capsule combined with salvia miltiorrhiza injection in the treatment of chronic renal failure. *Huaihai Medicine*.

[39] Peng W., Huang Y., Huang D. (2010). Clinical observation of kai Shi injection combined with Salvianolate injection in the treatment of 29 cases of chronic renal failure. *Hainan Medical Journal*.

[40] Liu H., Wang Z., Liu L. (2021). Clinical analysis of kai Shi injection combined with salvianolate in the treatment of chronic renal failure. *Modern Chinese Medicine Application*.

[41] Lv Y., Wang Y., Li W., Zhang L. (2006). Serum levels of NO, ET and IL-6 in patients with chronic renal failure with blood stasis syndrome and clinical study of the intervention effect of Leizhi Danshen tablets. *Proprietary Chinese Medicine*.

[42] Guowei W., Ding Y., Yang H., Li G. (2016). Efficacy of alprostadil combined with tanshinone II (A) in the treatment of elderly patients with chronic renal failure. *Journal of Guiyang College of Traditional Chinese Medicine*.

[43] Pang S. S. (2004). A summary of 20 cases of mild-moderate chronic renal failure treated by TCM catharsis combined with Salvia miltiorrhiza injection intravenous drip. *Hunan Chinese Medicine Review*.

[44] Chen C. (2016). Analysis of the efficacy of salvianolate injection in the treatment of chronic renal failure. *Journal of Practical Chinese Medicine*.

[45] Wang L. L. (2015). *Clinical Study of Salvianolate Combined with Valsartan in the Treatment of Hypertensive Nephropathy*.

[46] Zhi L., Gao Y., Wang W. (2016). Salvia miltiorrhiza phenolic acid salt combined renal failure more better curative effect observation of the treatment of chronic renal failure. *Journal of the China Hospital Drug Use Evaluation and Analysis*.

[47] Tong C., Lu J. (2012). Sodium ferulate combined with salvia miltiorrhiza injection in the treatment of chronic renal failure. *Chinese Medicine*.

[48] Xie S., Yang Y., Deng X. (2020). Effects of tanshinone II A sodium sulfonate injection combined with Bailing capsule on renal function and renal fibrosis in patients with chronic renal failure. *Hospital Drug Use Evaluation and Analysis in China*.

