# Supplementary material for: Efficacy and Safety of Salvia miltiorrhiza for Treating Chronic Kidney Diseases: A Systematic Review and Meta-Analysis
Source: Evid Based Complement Alternat Med. 2022 Jun 14;2022:2117433. doi: 10.1155/2022/2117433 (PMC9213127; doi:10.1155/2022/2117433)
Supplement: Supplementary Materials — Table S1: PRISMA 2020 Checklist. Table S2: search strategies. Table S3: certainty of Evidence evaluated by GRADEpro GDT. The original contributions presented in the study were included in the article/Supplementary Material; further inquiries could be directed to the first author. [file 2117433.f1.zip › 2117433.f1/Table S2.pdf]

Table S2 Search strategies

Database	Search terms
CENTRAL	#1 MeSH descriptor: [Kidney Diseases] explode all trees<br>#2 MeSH descriptor: [Renal Replacement Therapy] explode all trees<br>#3 MeSH descriptor: [Renal Insufficiency] explode all trees<br>#4 MeSH descriptor: [Renal Insufficiency, Chronic] explode all trees<br>#5 MeSH descriptor: [Renal Dialysis] explode all trees<br>#6 (dialysis):ti,ab,kw<br>#7 (hemofiltration):ti,ab,kw OR (haemofiltration):ti,ab,kw<br>#8 (hemodiafiltration):ti,ab,kw OR (haemodiafiltration):ti,ab,kw<br>#9 (CAPD):ti,ab,kw OR (CCPD):ti,ab,kw OR (APD):ti,ab,kw OR (PD):ti,ab,kw<br>#10 (ESRF):ti,ab,kw OR (ESKF):ti,ab,kw OR (ESRD):ti,ab,kw OR (ESKD):ti,ab,kw<br>#11 (predialysis):ti,ab,kw OR (pre - dialysis):ti,ab,kw<br>#12 ("kidney disease*"):ti,ab,kw OR ("renal disease*"):ti,ab,kw OR ("kidney failure"):ti,ab,kw OR (“renal failure”):ti,ab,kw<br>#13 (CKF):ti,ab,kw OR (CKD):ti,ab,kw OR (CRF):ti,ab,kw OR (CRD):ti,ab,kw<br>#14 (hemodialysis):ti,ab,kw OR (haemodialysis):ti,ab,kw<br>#15 MeSH descriptor: [Salvia miltiorrhiza] explode all trees<br>#16 (“Tan Seng”):ti,ab,kw<br>#17 (“Dan Shen”):ti,ab,kw<br>#18 (Danshen):ti,ab,kw<br>#19 (“Salvia, Chinese”):ti,ab,kw<br>#20 (“Chinese Salvi*”):ti,ab,kw<br>#21 (“Salvias, Chinese”):ti,ab,kw<br>#22 (“Salvia miltiorrhiz*”):ti,ab,kw<br>#23 (Tanshinone):ti,ab,kw<br>#24 (Salvianolate):ti,ab,kw<br>#25 #1 OR #2 OR #3 OR #4 OR #5 OR #6 OR #7 OR #8 OR #9 OR #10 OR #11 OR #12 OR #13 OR #14<br>#26 #15 OR #16 OR #17 OR #18 OR #19 OR #20 OR #21 OR #22 OR #23 OR #24<br>#27 #25 AND #26
Pubmed	#23 ((((((((((((((("Kidney Diseases"[Mesh]) OR ("Renal Replacement Therapy"[Mesh])) OR ("Renal Insufficiency"[Mesh])) OR ("Renal Insufficiency, Chronic"[Mesh])) OR ("Renal Dialysis"[Mesh])) OR (dialysis[Title/Abstract])) OR ((hemodialysis[Title/Abstract]) OR (haemodialysis[Title/Abstract]))) OR ((hemofiltration[Title/Abstract]) OR (haemofiltration[Title/Abstract]))) OR ((hemodiafiltration[Title/Abstract]) OR (haemodiafiltration[Title/Abstract]))) OR ((((CAPD[Title/Abstract]) OR (CCPD[Title/Abstract])) OR (APD[Title/Abstract])) OR (PD[Title/Abstract]))) OR ((((ESRF[Title/Abstract]) OR (ESKF[Title/Abstract])) OR (ESRD[Title/Abstract])) OR (ESKD[Title/Abstract]))) OR ((predialysis[Title/Abstract]) OR (pre - dialysis[Title/Abstract]))) OR (((("kidney disease*"[Title/Abstract]) OR ("renal disease*"[Title/Abstract])) OR ("kidney

	failure"[Title/Abstract])) OR ("renal failure"[Title/Abstract]))) OR ((((CKF[Title/Abstract]) OR (CKD[Title/Abstract])) OR (CRF[Title/Abstract])) OR (CRD[Title/Abstract]))) AND ((((("Salvia miltiorrhiza"[Mesh]) OR ((("Dan Shen"[Title/Abstract]) OR (Danshen[Title/Abstract])) OR ("Tan Seng"[Title/Abstract]))) OR (((("Chinese Salvi*"[Title/Abstract]) OR ("Salvia, Chinese"[Title/Abstract])) OR ("Salvias, Chinese"[Title/Abstract])) OR ("Salvia miltiorrhiz*"[Title/Abstract]))) OR (Salvianolate[Title/Abstract])) OR (Tanshinone[Title/Abstract]))) AND (randomized controlled trial[Publication Type] OR randomized[Title/Abstract] OR placebo[Title/Abstract])<br>#22 randomized controlled trial[Publication Type] OR randomized[Title/Abstract] OR placebo[Title/Abstract]<br>#21 randomized controlled trial[Publication Type] OR randomized[Title/Abstract] OR placebo[Title/Abstract]<br>#20 Tanshinone[Title/Abstract]<br>#19 Salvianolate[Title/Abstract]<br>#18 ((("Chinese Salvi*"[Title/Abstract]) OR ("Salvia, Chinese"[Title/Abstract])) OR ("Salvias, Chinese"[Title/Abstract])) OR ("Salvia miltiorrhiz*"[Title/Abstract])<br>#17 (("Dan Shen"[Title/Abstract]) OR (Danshen[Title/Abstract])) OR ("Tan Seng"[Title/Abstract])<br>#16 "Salvia miltiorrhiza"[Mesh]<br>#15 ((((((((((((("Kidney Diseases"[Mesh]) OR ("Renal Replacement Therapy"[Mesh])) OR ("Renal Insufficiency"[Mesh])) OR ("Renal Insufficiency, Chronic"[Mesh])) OR ("Renal Dialysis"[Mesh])) OR (dialysis[Title/Abstract])) OR ((hemodialysis[Title/Abstract]) OR (haemodialysis[Title/Abstract]))) OR ((hemofiltration[Title/Abstract]) OR (haemofiltration[Title/Abstract]))) OR ((hemodiafiltration[Title/Abstract]) OR (haemodiafiltration[Title/Abstract]))) OR ((((CAPD[Title/Abstract]) OR (CCPD[Title/Abstract])) OR (APD[Title/Abstract])) OR (PD[Title/Abstract]))) OR ((((ESRF[Title/Abstract]) OR (ESKF[Title/Abstract])) OR (ESRD[Title/Abstract])) OR (ESKD[Title/Abstract]))) OR ((predialysis[Title/Abstract]) OR (pre‐dialysis[Title/Abstract]))) OR (((("kidney disease*"[Title/Abstract]) OR ("renal disease*"[Title/Abstract])) OR ("kidney failure"[Title/Abstract])) OR ("renal failure"[Title/Abstract]))) OR ((((CKF[Title/Abstract]) OR (CKD[Title/Abstract])) OR (CRF[Title/Abstract])) OR (CRD[Title/Abstract]))<br>#14 (((CKF[Title/Abstract]) OR (CKD[Title/Abstract])) OR (CRF[Title/Abstract])) OR (CRD[Title/Abstract])<br>#13 ((("kidney disease*"[Title/Abstract]) OR ("renal disease*"[Title/Abstract])) OR ("kidney failure"[Title/Abstract])) OR ("renal failure"[Title/Abstract])<br>#12 (predialysis[Title/Abstract]) OR (pre‐dialysis[Title/Abstract])<br>#11 (((ESRF[Title/Abstract]) OR (ESKF[Title/Abstract])) OR (ESRD[Title/Abstract])) OR (ESKD[Title/Abstract])<br>#10 (((CAPD[Title/Abstract]) OR (CCPD[Title/Abstract])) OR (APD[Title/Abstract])) OR (PD[Title/Abstract])<br>#9 (hemodiafiltration[Title/Abstract]) OR (haemodiafiltration[Title/Abstract])

	#8 (hemofiltration[Title/Abstract]) OR (haemofiltration[Title/Abstract])<br>#7 (hemodialysis[Title/Abstract]) OR (haemodialysis[Title/Abstract])<br>#6 dialysis[Title/Abstract]<br>#5 "Renal Dialysis"[Mesh]<br>#4 "Renal Insufficiency, Chronic"[Mesh]<br>#3 "Renal Insufficiency"[Mesh]<br>#2 "Renal Replacement Therapy"[Mesh]<br>#1 "Kidney Diseases"[Mesh]
Embase	#19. ('kidney disease'/exp OR 'renal replacement therapy'/exp OR 'kidney failure'/exp OR 'chronic kidney failure'/exp OR 'hemodialysis'/exp OR (dialysis:ab,ti OR hemodialysis:ab,ti OR haemodialysis:ab,ti OR hemofiltration:ab,ti OR haemofiltration:ab,ti OR hemodiafiltration:ab,ti OR haemodiafiltration:ab,ti) OR (capd:ab,ti OR ccpd:ab,ti OR apd:ab,ti OR pd:ab,ti) OR (esrf:ab,ti OR eskf:ab,ti OR esrd:ab,ti OR eskd:ab,ti) OR (predialysis:ab,ti OR pre - dialysis:ab,ti) OR (ckf:ab,ti OR ckd:ab,ti OR crf:ab,ti OR crd:ab,ti) OR ('kidney disease*':ab,ti OR 'renal disease*':ab,ti OR 'kidney failure':ab,ti OR 'renal failure':ab,ti)) AND ('salvia miltiorrhiza'/exp OR ('tan seng':ab,ti OR 'dan shen':ab,ti OR 'chinese salvi*':ab,ti OR 'salvia, chinese':ab,ti) OR ('salvias, chinese':ab,ti OR 'salvia miltiorrhiz*':ab,ti OR 'danshen':ab,ti) OR ('salvianolate':ab,ti OR 'tanshinone':ab,ti)) AND (random:ab,ti OR 'placebo':ab,ti OR 'double-blind':ab,ti)<br>#18. random:ab,ti OR 'placebo':ab,ti OR 'double-blind':ab,ti<br>#17. 'salvia miltiorrhiza'/exp OR ('tan seng':ab,ti OR 'dan shen':ab,ti OR 'chinese salvi*':ab,ti OR 'salvia, chinese':ab,ti) OR ('salvias, chinese':ab,ti OR 'salvia miltiorrhiz*':ab,ti OR 'danshen':ab,ti) OR ('salvianolate':ab,ti OR 'tanshinone':ab,ti)<br>#16. 'salvianolate':ab,ti OR 'tanshinone':ab,ti<br>#15. 'salvias, chinese':ab,ti OR 'salvia miltiorrhiz*':ab,ti OR 'danshen':ab,ti<br>#14. 'tan seng':ab,ti OR 'dan shen':ab,ti OR 'chinese salvi*':ab,ti OR 'salvia, chinese':ab,ti<br>#13. 'salvia miltiorrhiza'/exp<br>#12. 'kidney disease'/exp OR 'renal replacement therapy'/exp OR 'kidney failure'/exp OR 'chronic kidney failure'/exp OR 'hemodialysis'/exp OR (dialysis:ab,ti OR hemodialysis:ab,ti OR haemodialysis:ab,ti OR hemofiltration:ab,ti OR haemofiltration:ab,ti OR hemodiafiltration:ab,ti OR haemodiafiltration:ab,ti) OR (capd:ab,ti OR ccpd:ab,ti OR apd:ab,ti OR pd:ab,ti) OR (esrf:ab,ti OR eskf:ab,ti OR esrd:ab,ti OR eskd:ab,ti) OR (predialysis:ab,ti OR pre - dialysis:ab,ti) OR (ckf:ab,ti OR ckd:ab,ti OR crf:ab,ti OR crd:ab,ti) OR ('kidney disease*':ab,ti OR 'renal disease*':ab,ti OR 'kidney failure':ab,ti OR 'renal failure':ab,ti)<br>#11. 'kidney disease*':ab,ti OR 'renal disease*':ab,ti OR 'kidney failure':ab,ti OR 'renal failure':ab,ti<br>#10. ckf:ab,ti OR ckd:ab,ti OR crf:ab,ti OR crd:ab,ti<br>#9. predialysis:ab,ti OR pre - dialysis:ab,ti<br>#8. esrf:ab,ti OR eskf:ab,ti OR esrd:ab,ti OR eskd:ab,ti<br>#7. capd:ab,ti OR ccpd:ab,ti OR apd:ab,ti OR pd:ab,ti<br>#6. dialysis:ab,ti OR hemodialysis:ab,ti OR haemodialysis:ab,ti OR

	hemofiltration:ab,ti OR haemofiltration:ab,ti OR hemodiafiltration:ab,ti OR haemodiafiltration:ab,ti<br>#5. 'hemodialysis'/exp<br>#4. 'chronic kidney failure'/exp<br>#3. 'kidney failure'/exp<br>#2. 'renal replacement therapy'/exp<br>#1. 'kidney disease'/exp
CNKI	#1. shen gong neng bu quan and man xing<br>#2. shen gong neng shuai jie and man xing<br>#3. man xing shen zang bing<br>#4. #1 or #2 or #3<br>#5. dan shen or dan shen tong or dan shen fen or dan shen duo fen suan<br>#6. sui ji<br>#7. #4 and #5 and #6
Wanfang Data	#1. shen gong neng bu quan and man xing<br>#2. shen gong neng shuai jie and man xing<br>#3. man xing shen zang bing<br>#4. #1 or #2 or #3<br>#5. dan shen or dan shen tong or dan shen fen or dan shen duo fen suan<br>#6. sui ji<br>#7. #4 and #5 and #6
SinoMed	#1. shen gong neng bu quan and man xing<br>#2. shen gong neng shuai jie and man xing<br>#3. man xing shen zang bing<br>#4. #1 or #2 or #3<br>#5. dan shen or dan shen tong or dan shen fen or dan shen duo fen suan<br>#6. sui ji or sui ji dui zhao shi yan<br>#7. #4 and #5 and #6
