# Supplementary material for: Efficacy and Safety of Salvia miltiorrhiza for Treating Chronic Kidney Diseases: A Systematic Review and Meta-Analysis
Source: Evid Based Complement Alternat Med. 2022 Jun 14;2022:2117433. doi: 10.1155/2022/2117433 (PMC9213127; doi:10.1155/2022/2117433)
Supplement: Supplementary Materials — Table S1: PRISMA 2020 Checklist. Table S2: search strategies. Table S3: certainty of Evidence evaluated by GRADEpro GDT. The original contributions presented in the study were included in the article/Supplementary Material; further inquiries could be directed to the first author. [file 2117433.f1.zip › 2117433.f1/Table S3.pdf]

Author(s): Wei Zhang
Question: Salvia miltiorrhiza or its extracts Salvianolate and Tanshinone compared to conventional treatment for chronic kidney disease

Certainty assessment							№ of patients		Effect		Certainty	Importance
№ of studies	Study design	Risk of bias	Inconsistency	Indirectness	Imprecision	Other considerations	Salvia miltiorrhiza or its extracts Salvianolate and Tanshinone	conventional treatment	Relative (95% CI)	Absolute (95% CI)		
**Scr**												
29	randomised trials	serious	not serious	not serious	not serious	none	1071	1011	-	SMD **0.6 SD lower** (0.79 lower to 0.41 lower)	⊕⊕⊕◯ Moderate	CRITICAL
**Ccr**												
13	randomised trials	serious	not serious	not serious	not serious	none	513	488	-	SMD **0.92 SD higher** (0.43 higher to 1.41 higher)	⊕⊕⊕◯ Moderate	CRITICAL
**GFR**												
3	randomised trials	serious	not serious	not serious	not serious	none	114	114	-	SMD **0.56 SD higher** (0.3 higher to 0.83 higher)	⊕⊕⊕◯ Moderate	CRITICAL
**BUN**												
26	randomised trials	serious	not serious	not serious	not serious	none	908	858	-	SMD **0.66 SD lower** (0.81 lower to 0.5 lower)	⊕⊕⊕◯ Moderate	IMPORTANT
**CysC**												
7	randomised trials	serious	not serious	not serious	not serious	none	162	153	-	SMD **5.16 SD lower** (14.84 lower to 4.53 higher)	⊕⊕⊕◯ Moderate	CRITICAL
**Effective Rate**												
15	randomised trials	serious	not serious	not serious	not serious	publication bias strongly suspected	474/558 (84.9%)	310/526 (58.9%)	**RR 0.30** (0.23 to 0.37)	**413 fewer per 1,000** (from 454 fewer to 371 fewer)	⊕⊕◯◯ Low	CRITICAL
**24h UPE**												
10	randomised trials	serious	not serious	not serious	not serious	none	344	331	-	SMD **0.7 SD lower** (1.21 lower to 0.19 lower)	⊕⊕⊕◯ Moderate	CRITICAL
**Time to initiation of dialysis**												
1	randomised trials	not serious	not serious	not serious	not serious	none	6/15 (40.0%)	14/15 (93.3%)	**RR 0.43** (0.23 to 0.81)	**532 fewer per 1,000** (from 719 fewer to 177 fewer)	⊕⊕⊕⊕ High	CRITICAL

Certainty assessment							№ of patients		Effect		Certainty	Importance
№ of studies	Study design	Risk of bias	Inconsistency	Indirectness	Imprecision	Other considerations	Salvia miltiorrhiza or its extracts Salvianolate and Tanshinone	conventional treatment	Relative (95% CI)	Absolute (95% CI)		
**Adverse effects**												
13	randomised trials	serious	not serious	not serious	not serious	publication bias strongly suspected	16/445 (3.6%)	24/439 (5.5%)	**RR -0.52** (-1.16 to 0.12)	**83 fewer per 1,000** (from 118 fewer to 48 fewer)	⨁⨁◯◯ Low	IMPORTANT
**ALB**												
4	randomised trials	serious	not serious	not serious	not serious	none	133	126	-	SMD **0.23 SD higher** (0.28 lower to 0.75 higher)	⨁⨁⨁◯ Moderate	NOT IMPORTANT
**TC**												
2	randomised trials	serious	not serious	not serious	not serious	none	62	61	-	SMD **0.53 SD lower** (0.88 lower to 0.17 lower)	⨁⨁⨁◯ Moderate	NOT IMPORTANT
**Hb**												
6	randomised trials	serious	not serious	not serious	not serious	none	422	402	-	SMD **0.42 SD higher** (0.13 higher to 0.71 higher)	⨁⨁⨁◯ Moderate	NOT IMPORTANT
**FIB**												
2	randomised trials	serious	not serious	not serious	not serious	none	79	72	-	SMD **0.79 SD lower** (1.12 lower to 0.46 lower)	⨁⨁⨁◯ Moderate	IMPORTANT
**CRP**												
3	randomised trials	not serious	not serious	not serious	not serious	none	140	130	-	SMD **0.56 SD lower** (0.93 lower to 0.19 lower)	⨁⨁⨁⨁ High	IMPORTANT

CI: confidence interval; **RR:** risk ratio; **SMD:** standardised mean difference
